# Toll-Like Receptors (TLRs) Expression in Contracted Capsules Compared to Uncontracted Capsules

**DOI:** 10.1007/s00266-019-01368-8

**Published:** 2019-04-01

**Authors:** Y. Bachour, M. J. P. F. Ritt, R. Heijmans, F. B. Niessen, S. P. Verweij

**Affiliations:** 1 Department of Plastic, Reconstructive and Hand Surgery, VU University Medical Center, De Boelelaan 1117, PO Box 7057, 1007 MB Amsterdam, The Netherlands; 2 Department of Medical Microbiology and Infection Control, VU University Medical Center, Amsterdam, The Netherlands

**Keywords:** Capsular contracture, Etiology, Breast implants, Breast augmentation, Toll-like receptors, Immunology

## Abstract

**Introduction:**

The etiology of capsular contracture after surgical implantation of breast implants remains unclear, but an important role is seen for the immune system. Toll-like receptors are immune receptors recognizing both pathogen-associated molecular patterns and damage-associated molecular patterns. The former are present on bacteria such as *Staphylococcus epidermidis* (bacteria earlier associated with capsular contracture), and the latter are released after (mechanical) stress. The aim of this study was to investigate the expression of TLRs 1–10 in relation to capsular contracture.

**Materials and Methods:**

Fifty consecutive breast capsules were collected during implant removal or replacement. The extent of capsular contracture was scored according to the Baker score. A sample specimen (0.5 cm^3^) was obtained from all tissues. cDNA was synthesized from isolated mRNA from the collected specimens. PCR analyses were conducted to test for cDNA presence and to quantify concentration. TLR1–10 expression was measured for each of the Baker scores separately and compared to all Baker scores.

**Results:**

Expression of all TLRs in all Baker scores was seen. TLR2 and TLR6 were more often present in contracted samples (Baker 3 or 4) compared to uncontracted samples (Baker 1 or 2) [Baker 2 vs. 3 (*p* = 0.034) and Baker 2 vs. 3 (*p* = 0.003), respectively]. None of the TLRs displayed a significantly higher expression in contracted capsules compared to uncontracted capsules.

**Conclusion:**

This study shows that TLR2 and TLR6 are more often expressed in contracted capsules compared to non-contracted capsules however not in higher concentrations.

**Level of Evidence III:**

This journal requires that authors assign a level of evidence to each article. For a full description of these Evidence-Based Medicine ratings, please refer to the Table of Contents or the online Instructions to Authors www.springer.com/00266.

## Background

Silicone breast implants have been widely used for breast augmentation or breast reconstruction after breast cancer [45]. Capsule formation is a normal foreign body reaction occurring around all implants [17, 34]. However, in selected cases this capsule formation around breast implants tends to progress, leading to capsular contracture. This complication presents with signs of hardening and disfiguring of the breast and is often painful [16]. Capsular contracture remains the most common complication after surgical implantation of breast implants with a prevalence ranging from 5 to 17% [18, 26] for breast augmentation and 19–25% [19, 31, 35] for breast reconstruction. The Baker score is used to clinically score the severity of capsular contracture, where Baker scores 1 and 2 represent normal capsules and Baker scores 3 and 4 represent contracted capsules [4].

The etiology of capsular contracture remains unknown [3]. An important observation is the role of the immune system in capsular contracture. All studies investigating the role of the immune system found a chronic inflammation in capsular contracture [13, 15, 22, 27, 37, 38, 49, 50]. A prominent role is seen for elements of innate immunity, such as activated macrophages, which, similar to polymorphonuclear neutrophils (PMNs), have nonspecific immune function. B1a and marginal zone B cells (MZB) are the first antibodies that initiate the process with participation of various interleukins as well as tumor necrosis factor alpha (TNF-α) [15, 27, 30, 38, 49]. This suggests that ongoing activity of the immune system is one of the causative factors in this multifactorial condition. Another important theory is that bacteria peri-prosthetically play a role in the etiology of capsular contracture. Some studies found associations between the presence of bacteria peri-prosthetically and capsular contracture [9, 12, 41, 51]. The bacteria cultured most often were *Staphylococcus* spp. To date, it is unknown whether bacteria trigger this inflammatory response.

**Fig. 1:**
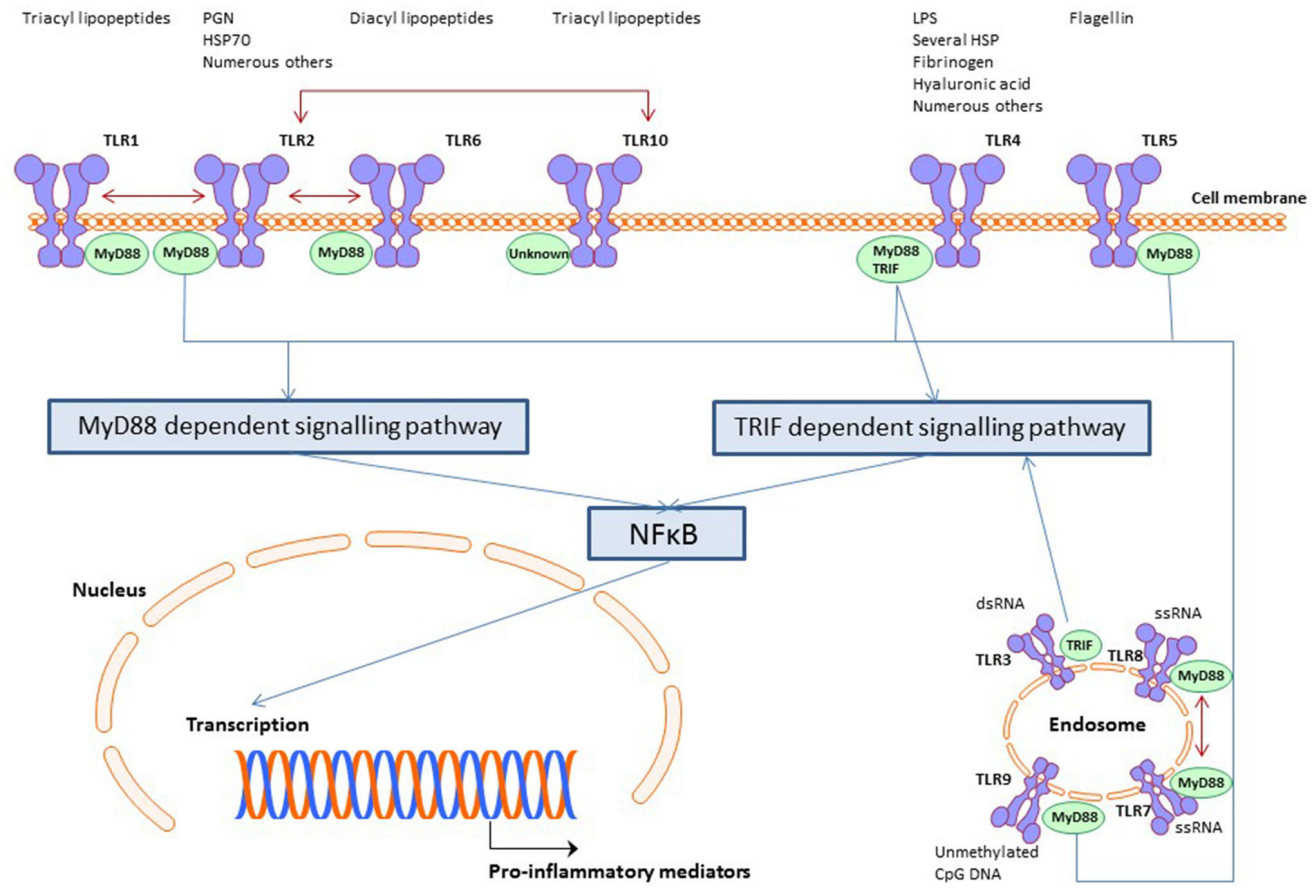
TLR cascade

Receptors on immune cells that recognize pathogens such as bacteria are Toll-like receptors (TLRs), previously known as modulins [10, 36]. These receptors recognize pathogenic surface ligands such as peptidoglycan (TLR2) and lipopolysaccharide (TLR4), or, for example, cytosine–guanine pairs intracellularly (TLR9), referred to as pathogen-associated molecular patterns (PAMPs). TLRs also recognize endogenous derived ligands such as oligosaccharides of hyaluronic acid and fibrinogen (TLR4) [10], and ligands that are generated during tissue damage (damage-associated molecular patterns (DAMPs) such as different heat shock proteins [6]. So far, 10 TLR members have been identified in humans. TLR1, TLR2, TLR4, TLR5, TLR6 and TLR10 are transmembrane proteins, while TLR3, TLR7, TLR8 and TLR9 are expressed intracellularly [7]. TLR3, TLR4, TLR5 and TLR9 are activated solely, while TLR 2 can form a heterodimer with TLR1 and TLR6. TLR7 and TLR8 are solely activated as a heterodimer [52]. See Fig. 1 for a simplified schematic representation. Because of their central role in the immune response, TLRs have become an important therapeutic target for the treatment of several diseases. For example, activators of TLRs (TLR agonists) are powerful immunostimulants due to their ability to drive innate and acquired immunity and are therefore used as adjuvants in vaccines [20].

Recognition of ligands by TLRs leads to intracellular signal transduction through the NF-κB pathway that in turn induces an immune cascade resulting in a host response, which is necessary to clear the pathogens [10]. If bacteria indeed play a significant role in the induction of capsular formation and contraction, it seems likely that TLRs are expressed in capsular contraction.

The aim of this study was therefore to investigate the expression of TLRs 1–10 in Baker 1–4 breast capsules.

## Materials and Methods

This was a cross-sectional study. Patient characteristics were retrospectively collected. Consecutive samples were obtained between 2014 and 2016 at the VU University Medical Center, Jan van Goyen Clinic and OLVG location West, in Amsterdam, the Netherlands. The local medical ethical committee approved this study (Study No. 2014.146). Written informed consent was provided by all participants.

### Sample Collection

Patients who underwent implant replacement or removal for any reason were included in this study. Patients who received radiotherapy were excluded from this study. The extent of capsular contracture was scored according to the Baker score [4] by two physicians who together reached an agreement on the score (see Table 1). Baker scores 1 and 2 were considered as uncontracted capsules, while Baker scores 3 and 4 were considered as contracted capsules. A sterile sample specimen (0.5 cm^3^) of the capsule was obtained from the removed capsule. The specimens were collected in sterile specimen containers followed by immediate snap-freezing in liquid nitrogen and stored at − 80 °C until further analysis. All samples were collected, stored and transported by the same physician (YB).

**Table 1:** Definition of baker grade I–IV

Baker grade	Breast description
I	Breast absolutely normal: soft to palpate, implant not palpable or visible
II	Minimal contracture: greater breast firmness than desired, implant palpable, but not visible
III	Moderate contracture; moderate breast firmness, implant easily palpable, breast deformity visible
IV	Severe contracture: severe breast firmness, hard, painful/tender, distortion marked, and sometimes a cold implant

### Laboratory Testing

Messenger RNA (mRNA) was isolated from the collected samples with the mRNA Catcher™ PLUS (InVitrogen) according to manufacturer’s protocol. Complementary DNA (cDNA) was synthesized from the isolated mRNA with the SuperScript™ III First-Strand Synthesis SuperMix for quantitative real-time polymerase chain reaction (qRT-PCR, Invitrogen) according to manufacturer’s protocol. In-house primers for the 10 TLR cDNAs were constructed. PCRs were performed with Thermo Fisher SYBR^®^ Select Master Mix (Cat. No.: 4472908): Real-time PCR conditions were 2 min at 95 °C (pre-incubation), followed by 40 cycles of 10 s at 95 °C, 10 s at 60 °C and 10 s at 72 °C (amplification). Outcomes were considered positive when the automated threshold (e.g., a fluorescent intensity above background level) was met.

### Data Analysis

Data were expressed as number (%) or mean (SD). The Chi-square test was used for discrete datasets (negative and positive outcomes), while the Mann–Whitney *U* test was used for continuous data (concentration of cDNA), and *p* values less than 0.05 were considered statistically significant. Due to the limited number of investigated genes, correction for multiple testing was not performed to prevent underestimation of possible associations [40]. Statistical tests were performed using SPSS 22.0 (IBM SPSS Statistics for Windows, version 22.0. IBM Corp., Armonk, NY, USA).

## Results

### Subjects and Implant Characteristics

This study included 50 samples obtained form 26 patients. The primary indication for breast implantation was in most cases cosmetic (94%) and in 6% for reconstructive surgery after breast cancer. The mean age at the present surgery was 46 years (SD 12.0 years). The Baker score was grade 1 or 2 in 28 (56%) cases [Baker 1, *n* = 12 (24%), Baker 2, *n* = 16 (32%)] and grade 3 or 4 in 22 (44%) cases [Baker 3, *n* = 10 (20%), Baker 4, *n* = 12 (24%)]. Implants were implanted for a mean of 11 years (SD 5.7 years). The implantation duration for the Baker score was as follows: Baker 1: 6.6 years (SD 5.0 years), Baker 2: 11.5 years (SD 5.5 years), Baker 3: 8.8 years (SD 4.9 years) and Baker 4: 15.3 years (SD 4.0 years). This was significantly higher for higher Baker scores: Baker 1 versus Baker 2 (*p* = 0.033), Baker 1 versus Baker 4 (*p* = 0.002) and Baker 3 versus Baker 4 (*p* = 0.017).

### Toll-Like Receptors Expression

Data for the TLR expression for Baker 1–4 are presented in Tables 2 and 3. Expression of all TLRs in all Baker scores was observed. There was a significant difference in expression for TLR2 [Baker 1 vs. 2 (*p* = 0.008) and Baker 2 vs. 3 (*p* = 0.034)], TLR6 [Baker 2 vs. 3 (*p* = 0.003)] and TLR8 [Baker 1 vs. 2 (*p* = 0.015), Baker 1 vs. 4 (*p* = 0.035)]. TLR2 (Baker 2 vs. 3, *p* = 0.034) and TLR6 (Baker 2 vs. 3, *p* = 0.003) were more often expressed in contracted capsules compared to uncontracted capsules. There was a trend toward a difference for TLR 3 (Baker 1 vs. 4, 58.3% vs. 91.7%, *p* = 0.059) and TLR 10 (Baker 2 vs. 3, 31.3% vs. 70.0%, *p* = 0.054).

**Table 2:** Number of negative and positive tests in Baker 1–4 breast capsules using the Chi-square test

TLR	Baker 1 (*n* = 12)		Baker 2 (*n* = 16)		Baker 3 (*n* = 10)		Baker 4 (*n* = 12)		*p* Value
	Negative n (%)	Positive n (%)	Negative n (%)	Positive n (%)	Negative n (%)	Positive n (%)	Negative n (%)	Positive n (%)	
1	9 (75.0)	3 (25.0)	12 (75.0)	4 (25.0)	7 (70.0)	3 (30.0)	7 (58.3)	5 (41.7)	NS
2	6 (50.0)	6 (50.0)	15 (93.7)	1 (6.3)	6 (60.0)	4 (40.0)	6 (50.0)	6 (50.0)	S*
3	5 (41.7)	7 (58.3)	5 (31.3)	11 (68.7)	4 (40.0)	6 (60.0)	1 (8.3)	11 (91.7)	NS
4	6 (50.0)	6 (50.0)	10 (62.5)	6 (37.5)	5 (50.0)	5 (50.0)	5 (41.7)	7 (58.3)	NS
5	6 (50.0)	6 (50.0)	9 (56.3)	7 (43.7)	5 (50.0)	5 (50.0)	2 (16.7)	10 (83.3)	NS
6	4 (33.3)	8 (66.7)	11 (68.7)	5 (31.3)	1 (10.0)	9 (90.0)	3 (25.0)	9 (75.0)	S**
7	2 (16.7)	10 (83.3)	4 (25.0)	12 (75.0)	4 (40.0)	6 (60.0)	4 (33.3)	8 (66.7)	NS
8	2 (16.7)	10 (83.3)	10 (62.5)	6 (37.5)	5 (50.0)	5 (50.0)	7 (58.3)	5 (41.7)	S***
9	1 (8.3)	11 (91.7)	4 (25.0)	12 (75.0)	1 (10.0)	9 (90.0)	2 (16.7)	10 (83.3)	NS
10	5 (57.1)	7 (42.9)	11 (68.7)	5 (31.3)	3 (30.0)	7 (70.0)	3 (25.0)	9 (75.0)	NS

Outcomes were considered positive when the automated threshold (e.g., a fluorescent intensity above background level) was met

*NS* not significant, *S* significant

*Baker 1 versus 2 (*p* = 0.008), Baker 2 versus 3 (*p* = 0.034)

**Baker 2 versus 3 (*p* = 0.003)

***Baker 1 versus 2 (*p* = 0.015), Baker 1 versus 4 (*p* = 0.035)

**Table 3:** Mean concentration cDNA (copies/µlcDNA) in Baker 1–4 breast capsules using the Mann–Whitney *U* test

TLR	Baker 1 (*n* = 12) Mean (SD)	Baker 2 (*n* = 16) Mean (SD)	Baker 3 (*n* = 10) Mean (SD)	Baker 4 (*n* = 12) Mean (SD)	*p* Value
1	0.8 (1.9)	0.1 (0.2)	0.3 (0.5)	0.6 (1.2)	NS
2	0.3 (0.5)	0.6 (2.4)	0.3 (0.7)	1.0 (2.1)	S*
3	2.6 (2.7)	9.7 (27.6)	4.5 (4.0)	8.4 (7.8)	NS
4	3.2 (5.2)	2.0 (5.4)	1.2 (1.5)	2.2 (2.5)	NS
5	1.7 (2.4)	1.9 (6.3	1.1 (1.6)	2.6 (2.5)	NS
6	3.7 (4.2)	3.6 (11.7)	3.0 (3.0)	3.6 (4.0)	S**
7	1.9 (1.6)	1.9 (5.0)	2.0 (2.9)	1.8 (3.1)	NS
8	0.6 (0.6)	0.2 (0.4)	0.3 (0.4)	0.2 (0.4)	S***
9	4.3 (3.8)	5.3 (10.7)	3.4 (3.3)	3.1 (3.0)	NS
10	2.1 (6.5)	2.0 (6.5)	0.9 (1.3)	1.1 (1.5)	NS

*NS* not significant, *S* significant

*Baker 1 versus 2 (*p* = 0.017)

**Baker 1 versus 2 (*p* = 0.049), Baker 2 versus 3 (*p* = 0.021)

***Baker 1 versus 2 (*p* = 0.018)

As can be seen in Table 3, there was a significant difference of the mean concentration of TLR2 (Baker 1 vs. 2, *p* = 0.017), TLR6 (Baker 1 vs. 2, *p* = 0.049 and Baker 2 vs. 3, *p* = 0.021) and TLR8 (Baker 1 vs. 2, *p* = 0.018). A trend was seen for a higher expression of TLR2 (Baker 2 vs. 3, *p* = 0.056) and TLR8 (Baker 1 vs. 4, *p* = 0.058). None of the TLRs displayed a significantly higher expressed in contracted capsules compared to uncontracted capsules.

## Discussion

The aim of this study was to investigate the expression of TLRs in breast capsules to further elucidate the immune cascade leading to capsular contracture. The current study found a significantly higher expression of TLR2 and TLR6 in contracted capsules (Baker 3 or 4) in comparison with normal capsules (Baker 1 or 2). None of the TLRs displayed a significantly higher expressed in contracted capsules compared to uncontracted capsules.

Although not completely understood, an ongoing immune response is observed in capsular contracture [13, 15, 22, 27, 37, 38, 49, 50]. Activated macrophages and monocytes secrete cytokines and chemokines such as IL-8 and TNF-α which induce a pro-inflammatory immune response [15, 27, 38, 49]. Hereafter, activation of NK cells and fibroblast is seen [14, 39]. It is unknown which pathogen or signaling factor induces this immune response. However, there is evidence that bacteria in situ might be involved in the etiopathogenesis of capsular contracture. The presence of bacteria has been described in several studies [9, 12, 41, 51]. Multiple studies have found a higher number of bacteria in contracted capsules compared to normal breast capsules [9, 12, 41, 51]. In these studies, the most common cultured bacteria were coagulase-negative staphylococci such as *Staphylococcus epidermidis.* Other frequently cultured bacteria were *Staphylococcus aureus* and *Propionibacterium acnes*. Although several studies found associations between the presence of bacteria, conflicting data exist [1]. Nonetheless, it has never been investigated whether these bacteria induce an immune response via, for example, TLR activation in relation to capsular contracture.

*Staphylococcus epidermidis* is a commensal gram-positive bacterium which contains phenol-soluble modulin ligands [10] on its surface which is recognized by TLR2 receptors. In the current study, TLR2 was significantly more often expressed in Baker 3 (40% positive) capsules compared to Baker 2 (6.3% positive) capsules. There was, however, no difference in the mean concentration of cDNA between contracted and uncontracted capsules. *Staphylococcus aureus* and *Propionibacterium acnes* are also commensal skin bacteria, but with peptidoglycan (PGN) ligands on their surface which is also recognized by TLR2 [48]. If bacterial infection with *S. epidermidis*, *S. aureus* or *P. acnes* would have activated an immune reaction, much higher TLR2 expression would have been expected in the capsules. In our study, there was also a significant higher expression of TLR6 (Baker 2 vs. Baker 3, 31.3% vs. 90%) observed in contracted capsules compared to non-contracted capsules inversely with a significant higher mean concentration for uncontracted (Baker 2) versus contracted capsules (Baker 3). TLR6 can form a heterodimer with TLR2 [47]. Together, they function as a receptor for (gram-positive) recognition of diacyl lipopeptides ligands on, for example, *Mycoplasma* spp [10]. It is, however, unlikely that a *Mycoplasma* infection occurs around breast implants. This would result in a fulminant clinical infection which has not been reported by our patients. Moreover, all capsules have been tested for microbial presence in our former study finding no *Mycoplasma* spp. on the capsules [1]. In that study, we investigated the presence of bacteria on the same set of capsules using a highly sensitive PCR method. Here, we observed that breast capsules are generally sterile. Moreover, no association was found between the presence of bacteria and the Baker score.

TLR8 was significantly more often expressed in Baker 1 capsules compared to Baker 2 and Baker 4 capsules. TLR8, which is expressed intracellularly, is known to interact with single-stranded RNA from viruses, and small synthetic compounds [10]. Our former study on the same capsules, however, did not find any viruses in the capsules [1].

None of the TLRs displayed a significantly higher expressed in contracted capsules compared to uncontracted capsules. This is thought to be less important since TLRs expression does not correlate well with the TLRs on the membrane (or intracellularly), and it therefore just a measurement for expression.

Only one study investigated the role of TLR expression in breast capsules. Segreto et al. [42], investigated the expression of TLR4 and its relationship with estrogen receptor expression. They found TLR4 expression in fibroblasts and myofibroblasts with a positive correlation with estrogen receptor-β expression in capsular contracture. They postulate that estrogen receptor-β increases the TGF-β production which is a stimulus involved in myofibroblast differentiation and contraction. This study suggests a role for hormonal signaling in the etiology of capsular contracture. TLR4 is, however, mostly known to be one of the major receptors for the recognition of lipopolysaccharide (LPS), a ligand on Gram-negative bacteria [10, 29]. Upregulation in contracted capsules as observed by Segreto et al. [42] could suggest that TLR4 is activated by the presence of bacteria. It would have been valuable if they would also have investigated bacterial presence on their capsules to strengthen this theory. In our study, we also observed upregulation of TLR4 in all four groups. However, no differences in expression of concentrations of cDNA were observed between the four investigated groups. Our follow-up time was much longer (a mean of 11 years in comparison with a mean of 1 year by Segreto et al.), with the possibility of a decline of TLR expression over time.

Since upregulation of all TLRs is observed after a mean implant duration of 11 years, it seems likely that an ongoing stimulus activates TLR expression. It is expected that after 11 years bacteria are resolved by the immune system, as has been confirmed in our sterile samples [1], making PAMPs an unlikely factor for the observed TLR expression. DAMPs, however, can be generated over a long period of time. Different types of DAMPs, such as heat shock proteins (HSPs), are released under conditions of stress or tissue injury such as traumatic lesions, burns and oxidative stress [11, 28]. The major HSPs that are generated during tissue damage are HSP 47 and 70 [24, 46]. These HSPs are then recognized by pattern recognition receptors such as TLRs and contribute to an immune response causing tissue damage [5]. HSP 70, for example, is recognized by TLR2 [32, 53]. HSP 47 and 70 have been associated with different fibrotic diseases such as keloids, lung fibrosis and cardiac hypertrophy [21, 23, 33]. Therefore, it seems not unlikely that the same process occurs in capsular contracture. We hypothesize that HSPs are released as a consequence of mechanical friction of the implant in the surrounding tissue causing cell injury. The release of these molecules might cause an expression of TLR2, as has been found to be significantly higher in our contracture group, leading to an immune response causing tissue damage. This hypothesis may explain the high rates of capsular contracture in patients with textured or smooth implants [2, 8, 25] which can move freely in the surrounding tissue and cause mechanical stress in contrast to polyurethane implants [43] that usually grow within the surrounding tissue and cause minimal mechanical stress. Further research, however, should provide more insight in the release of DAMPs in capsular contracture.

To our knowledge, this is the first study investigating expression of the full human TLR set in the context of capsular contracture. There are nonetheless several limitations to the present study. First of all, we included mostly female patients who underwent the primary surgical procedure for cosmetic reasons, but we also included two female patients who underwent breast reconstruction as a primary indication. Secondly, the implant duration in our study was quite long. It is possible that bacteria or any other factor have triggered initial TLR expression, but that this expression has declined over time as bacteria were cleared by the host immune system. There is also a significant difference between the Baker scores in implant duration. This difference alone could be an explanation for the difference between the groups. Also, data on “time to complaint” are not available since it was difficult for patients to mention the start of their complaints. This would have been beneficial since bacterial activity and immune response would be different at different time intervals independent of capsular contracture. Third, TLR expression was measured based on mRNA expression, which is rapidly degradable. Therefore, samples were immediately collected and transported in liquid nitrogen to prevent degradation. Samples were placed in dry ice during laboratory testing as much as possible. Although samples were handled with care during transportation and laboratory testing, it is inevitable that some of the mRNA degraded during sample transportation and laboratory testing. This may have influenced the results and makes it difficult to reproduce this test even though standardized assays were used. Fourth, there are also routes other than TLRs activation by which the immune system can be activated [44]. Staphylococcus aureus and group A streptococci, for example, produce superantigens. These superantigens first bind to MHC class II and then to T cell receptors inducing a signaling pathway resulting in the production of several cytokines. This mechanism bypasses the TLR route. In such a case, the TLR mechanism is less relevant in the pathogenesis of capsular contracture. Fifth, the Baker score was used to grade the degree of contracture. Patients with some palpable firmness and pain were therefore automatically categorized as Baker 4. Because this scale is not completely objective, some error might have been introduced.

More and larger studies are needed to confirm our findings. First, it would be valuable to perform a follow-up study in which samples with a lower implant duration were collected to test the theory that there could be a decline in TLR expression over time. Furthermore, it should be investigated whether DAMPs indeed are released in breast and capsular tissue. Also, intracellular signaling after TLR activation occurs through the MyD88, MAL, TRIF, TRAM or TAK-1 pathway [10]. Further research is needed to investigate which of these intracellular signaling pathways is activated in capsular contracture. This could give more insight into the exact immune cascade leading to capsular contracture. Lastly, other receptors on immune cells have been identified and should be investigated to give more insight into the activation and signaling pathways leading to capsular contracture.

## Conclusion

The present study shows that TLR2 and TLR6 are more often expressed in contracted capsules compared to non-contracted capsules. We suggest that bacteria are not per se involved in the activation of an immune cascade leading to capsular contracture, but hypothesize that other ligands, such as DAMPs, might be involved.
